# Multicolor melting curve analysis discloses high carrier frequency of hearing loss‐associated variants among neonates in Jiangsu province

**DOI:** 10.1002/mgg3.2384

**Published:** 2024-02-06

**Authors:** Yi Liu, Yuanyuan Zhang, Jue Wang, Shengnan Song, Huiyan Wang, Qian Meng, Yuan Zhan, Yetao Xu, Lizhou Sun

**Affiliations:** ^1^ Department of Obstetrics & Gynecology The First Affiliated Hospital of Nanjing Medical University Nanjing Jiangsu China; ^2^ Department of Obstetrics & Gynecology Changzhou Maternity and Child Health Care Hospital Changzhou Jiangsu China; ^3^ Department of Obstetrics & Gynecology Lianyungang Maternity and Child Health Hospital Lianyungang Jiangsu China

**Keywords:** genetic screening, hearing loss, hearing screening, melt‐curve analysis, neonate

## Abstract

**Background:**

Genetic disorders ascribe to half of cases of congenital hearing loss. Hearing screening is significant in detecting hearing loss (HL) but weak at diagnosis, which can be complemented by genetic screening.

**Methods:**

To find a feasible method to accomplish genetic screening and evaluate its advantage when combined with hearing screening, between 1 January 2022, and 10 December 2023, we performed an observational cohort study based on 2488 neonates from the Han population at three hospitals in Jiangsu province. Genetic screening for 20 variants in four common HL‐associated genes by multicolor melting curve analysis (MMCA) and hearing screening were offered concurrently to all participants.

**Results:**

In total, 170 (6.8%) of 2488 eligible neonates were detected at least one variant and among them, the proportion of referral was higher (*p* < 0.05). Genetic screening combined with hearing screening was associated with a 25.0% increase (2 of 8) in discovering cases of diagnosed hearing loss that were missed by hearing screening.

**Conclusion:**

This study suggests that genetic screening combined with hearing screening by MMCA is effective at finding potential HL cases and practical to be validated in other places.

## Graphical abstract

Genetic screening by multicolor melting curve analysis combined with hearing screening is profit to diagnosis of hearing loss of neonates.

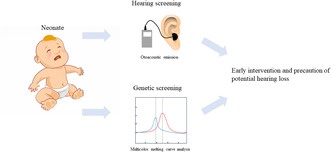

## 1 INTRODUCTION

Hearing loss (HL) is a prevalent human disorder with complex etiologies. Globally, approximately two out of 1000 neonates experience permanent HL. In about half of these cases, HL can be attributed to genetic factors (Fang et al., 2020; Korver et al., 2017; Yuan et al., 2020). In China, the prevalence of hereditary HL is similar, with approximately 20,000–30,000 newly affected children each year (Wang, Xiang, et al., 2019; Yuan et al., 2020). Due to the widespread implementation of universal newborn hearing screening (UNHS) and improvements in sanitation conditions, the proportion of HL attributed to infections in China is decreasing. Consequently, more children with HL can receive timely interventions (Fang et al., 2020; Shearer et al., 2019; Tordrup et al., 2022). However, approximately one‐fourth of HL cases without a clear etiology may go undiagnosed and what potentially impute to it is that some of them ascribing to HL‐associated variants manifest as delayed‐onset cases or acquired ones induced by factors such as ototoxic drugs or head impact (Lieu et al., 2020; Minami et al., 2013; Wang, Xiang, et al., 2019; Zhu et al., 2021).

The majority of genetic HL cases in China were attributed to variants in four specific genes: gap junction beta‐2 gene (*GJB2*; OMIM accession number: 121011; NC_000013.11 GRCh38.p14), solute carrier family 26 member 4 gene (*SLC26A4*; OMIM accession number: 605646; NC_000007.14 GRCh38.p14), mitochondrially encoded 12S RNA (*MT‐RNR1*; OMIM accession number: 561000; NC_012920.1 GRCh38.p14), and gap junction beta‐3 gene (*GJB3*; OMIM accession number: 603324; NC_000001.11 GRCh38.p14) (Fu et al., 2019). However, it is important to note that the distributions of HL‐associated variants may differ across regions and ethnic groups (Zhang et al., 2023). In China, certain provinces and regions started implementing genetic screening together with hearing screening initiatives from 2006 (Chen et al., 2015; Dai et al., 2019; Hao et al., 2018; Peng et al., 2016; Zhu et al., 2021). In Jiangsu province, genetic screening initiatives were implemented later and limited studies conducted in Jiangsu revealed here a hyperendemic area of genetic HL compared to other provinces (Chen et al., 2015; Hao et al., 2018; Wen et al., 2020).

To screen for HL‐associated variants, several reliable methods have been adopted, such as microarray assay, next‐generation sequencing, and flight mass spectrometry. However, the use of microarray‐based approaches is hindered by their prohibitive cost, lengthy processing time, and requirement for sophisticated laboratory settings, which limit their implementation in underdeveloped areas. In contrast, the multicolor melting curve analysis method has shown promise in detecting infectious and congenital diseases due to its accuracy, convenience, and cost‐effectiveness in terms of time and expenses (Huang et al., 2016; Xu et al., 2019). The objective of this project is to promptly detect HL or potential HL in neonates and to develop a strategy for implementing hearing screening along with genetic screening in Jiangsu province and assess the feasibility of promoting this screening approach in other regions.

## 2 MATERIALS AND METHODS

### 2.1 Study design and ethics

We conducted simultaneous screenings for both deafness genes and hearing ability to assess their agreement rate and analyze the epidemiology of deafness genes in Jiangsu province. This observational and prospective study was conducted in multiple centers to ensure its representativeness. The participating centers were as follows: Nanjing (The First Affiliated Hospital of Nanjing Medical University); Changzhou (The Maternal and Child Health Hospital of Changzhou); and Lianyungang (The Maternal and Child Health Hospital of Lianyungang). At each center, hearing screening, hearing rescreening, collection of samples (dried blood spots), and clinical follow‐up were conducted by trained nurses and audiologists. Experiments of genetic screening were finished in Nanjing (the middle center) after sample collection. This study was approved by the ethics committees of Ethics Committee of Jiangsu province Hospital affiliated with Nanjing Medical University.

**FIGURE 1:**
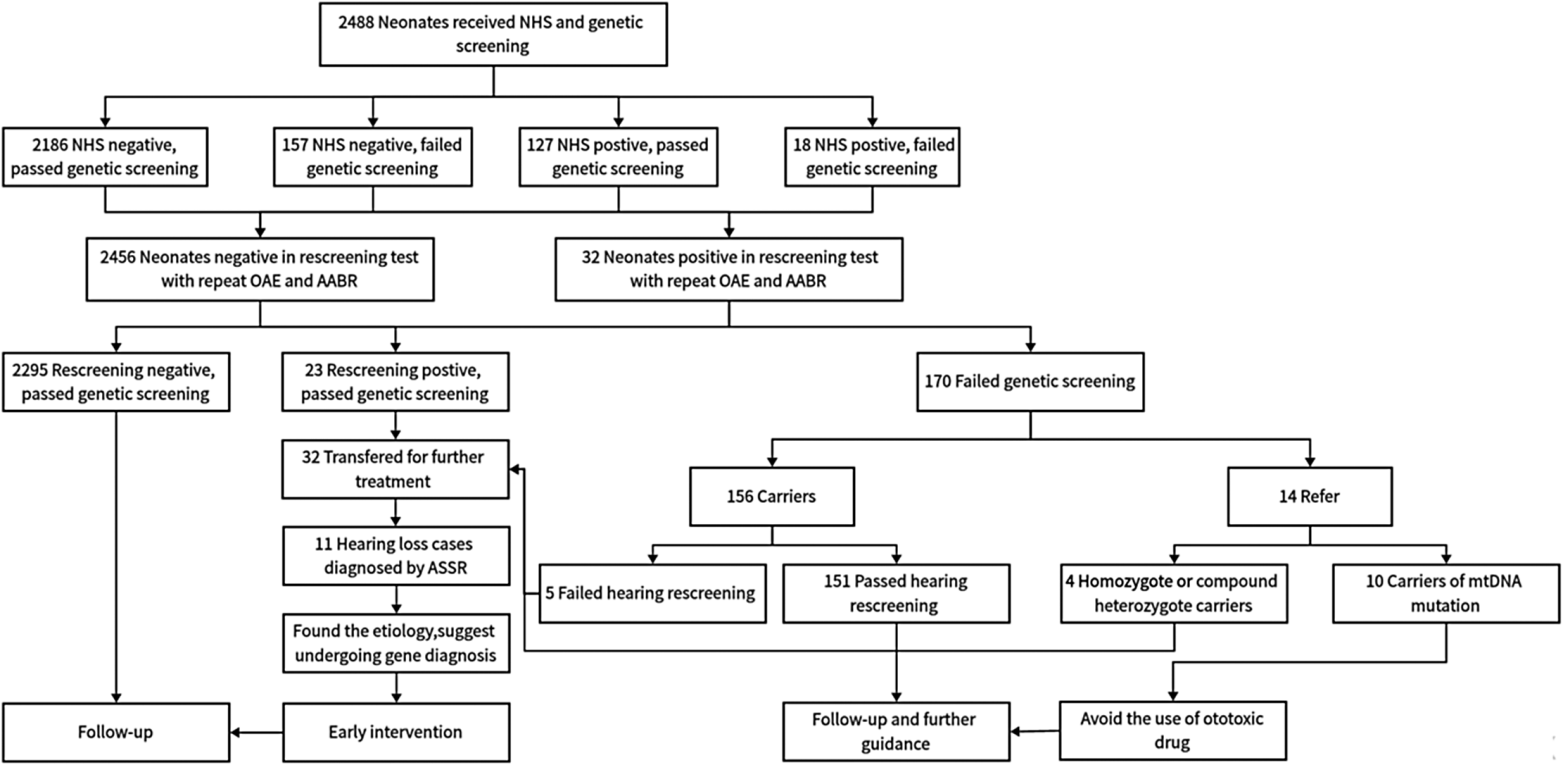
Overview of hearing screening combined with genetic screening program. AABR, automated auditory brainstem response; AASR, auditory steady‐state response; NHS, newborn hearing screening; OAE, otoacoustic emission.

We established selection criteria to minimize false‐positive results. These criteria included: (1) Neonates or pregnant women diagnosed with cytomegalic inclusion disease (detection of immunoglobulin M in the serum of pregnant women or neonates, and cytomegalovirus in the urine of neonates within 3 weeks after birth); (2) Neonates with clearly identified physical causes of deafness; and (3) Neonates whose health conditions did not allow for further examinations. The procedure consisted of three stages: stage 1, before discharge (within 3 days after birth), hearing screening and genetic screening were offered concurrently to the included neonates; stage 2, about 42 days after birth, all neonates went through hearing rescreening; and stage 3, the infants who did not pass hearing rescreening were referred and at 3–6 months of age, hearing tests were scheduled for diagnosis and further treatment. A flowchart (Figure 1) depicts the procedure of this study.

To ensure ethical standards, the participation of neonates required informed consent from their guardians. All recruited neonates received both newborn hearing screening and genetic screening free of charge. The government funded the hearing screening, while research project foundations sponsored the genetic screening. Clinic information, including sex, nationality, date of birth, sampling date, neonatal diseases (if any), history of inbreeding (if any), family history of HL (if any), and medication history during the neonatal period, were collected through medical records or telephone follow‐up.

### 2.2 Sample data

Between January 2022 and October 2022, a total of 2506 neonates in Jiangsu province were initially included in the study. Eighteen neonates were subsequently excluded for assorted reasons. Specifically, seven neonates' parents declined to participate in the follow‐up of hearing screening. Two neonates had insufficient DNA for analysis due to extraction issues or inappropriate storage methods, and after communication, parents of them refused to collect their child's blood sample for resumption. After hearing and genetic screening, nine neonates missed follow‐up for leaving invalid contact information. Totally, 2488 neonates from the Han population in China completed the study and underwent follow‐up procedures, which consisted of 1235 males and 1253 females.

### 2.3 Hearing screening

Hearing screening and diagnosis follow the guidelines of the UNHS (2018). Each newborn underwent the newborn hearing screening (NHS) using otoacoustic emission (OAE) within 24–48 h after birth. The second hearing screening included rescreening with automated auditory brainstem response (AABR) at 42 days after birth. If the newborn failed the rescreening, further hearing diagnosis would be applied with AABR and auditory steady‐state response (ASSR). The severity of HL was classified into four categories: mild (26–40 dB), moderate (41–60 dB), severe (61–80 dB), and profound (>81 dB).

### 2.4 Genetic screening

We selected 20 variants, which were found to have high diagnostic value among patients with HL or reported to cause HL in other countries but in lack of detailed information reflecting their prevalence in China (Danilchenko et al., 2022; Xie et al., 2021). The selected variants included: *GJB2* (c.35delG, c.176_191del16, c.235delC, c.299_300delAT, c.167delT), *GJB3* (c.538C>T, c.547G>A), *MT‐RNR1* (m.1494C>T, m.1555A>G), *SLC26A4* (c.919‐2A>G, c.1174A>T, c.1226G>A, c.1229C>T, c.1707+5G>A, c.1975G>C, c.2168A>G, c.2027T>A, c.2162C>T, c.749T>C, c.754T>C, c.2027T>A).

**FIGURE 2:**
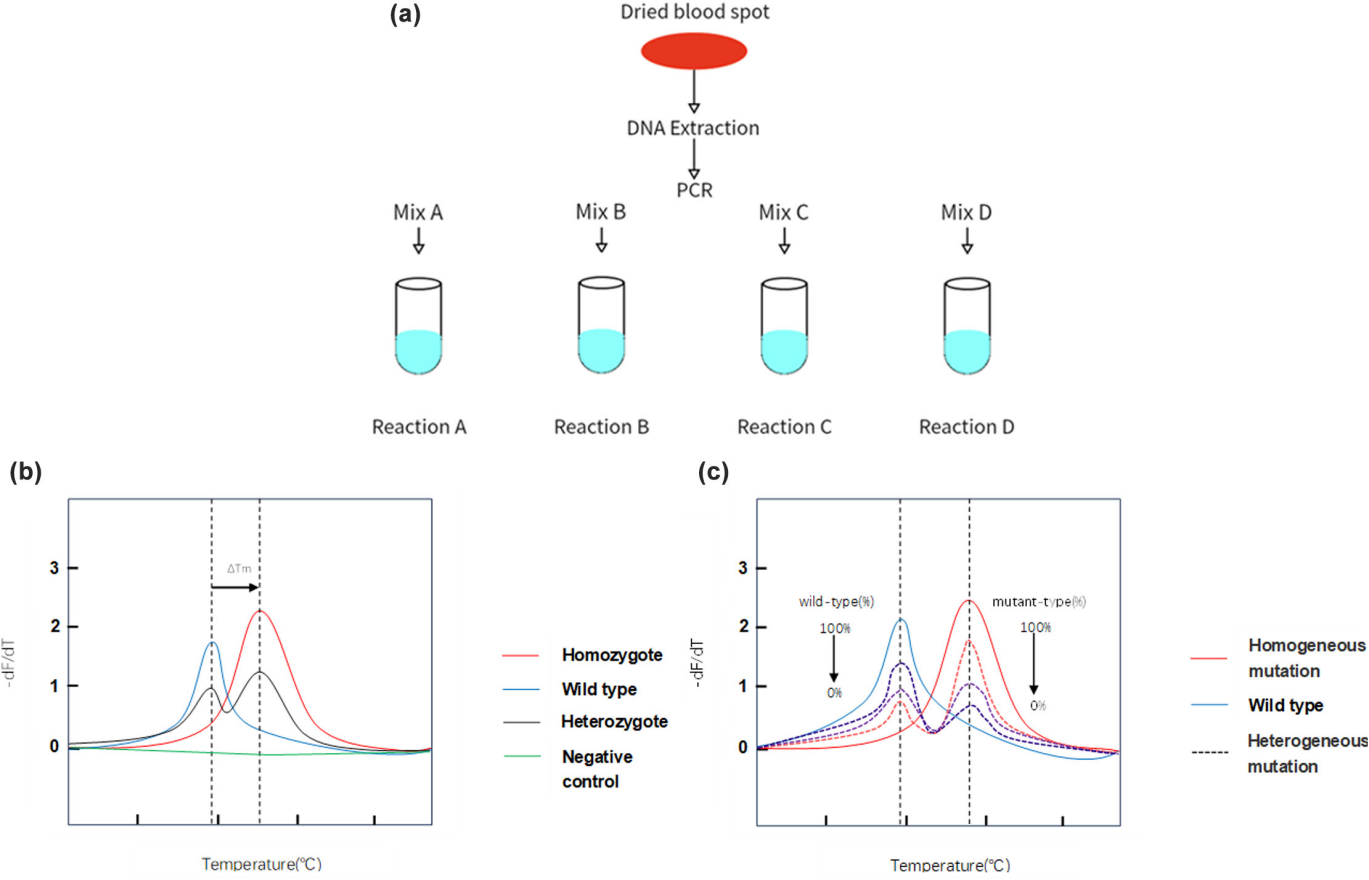
Step of MMCA assay for the detection of HL‐associated mutations. (a) Genomic DNA was extracted from blood samples and then added into four PCR reactions (each reaction contains the primers and the detection probes labeled four different labeled fluorophores of FAM, HEX, ROX, and CY5). (b) Red line and blue line represent the melting peaks of homozygous mutation type and wild type, besides that the black line means heterozygous mutation type, which has two melting peaks same with homozygous mutation type and wild type. (c) Red line and blue line show the melting peaks of homogeneous mutation and wild type. Dashed line means heterogeneity mutation with two melting peaks and lines in different colors indicating higher or lower peak value of −dF/dT mean different ratio of mitochondrion with mutant‐type *MT‐RNR1*.

Dried blood spots were collected from the heels of infants within 24–48 h after birth and DNA samples were extracted from them with a nucleic acid extraction kit (Zeesan Biotech, Xiamen, China) and the Lab Aid 824 automatic DNA extraction system (Zeesan Biotech, Xiamen, China). The above‐mentioned variants were detected using MMCA and its process and interpretation of results were presented in Figure 2a. The extracted DNA was added to four PCR tubes with primers, and PCR amplification and melting curve analysis were performed as programmed. Graphic output with melting temperature (Tm) values was automatically generated upon completion and difference in Tm within standard deviation. Cycle, threshold, temperature, and Tm values were analyzed and plotted by program. In Figure 2b, the genotypes of *GJB2*, *MT‐RNR1*, *SLC26A4*, and *GJB3* could be detected by the values of Tm and ΔTm. Unlike other variants that were confirmed by the difference in Tm from the wild type, the melting curve of the two mitochondrial variants (m.7444G>A, m.7445A>G) varied based on the proportion of mitochondria with the corresponding variant in Figure 2c. In Table 1, variants detected by corresponding reaction and fluorescence dye were shown in upper table. Tm. difference (ΔTm) of 20 variants with wild‐type and its three times standard deviation (3SD) were presented in lower table. ΔTm was much larger than SD, which made the results discriminable and convincing. Results of genetic screening were categorized as negative (wild type), carrier (heterozygote or compound heterozygous variant), and refer (homozygote or biallelic variant of *GJB2*, *SLC26A4*, and *GJB3*).

**TABLE 1:** Calibrated Tm values of 20 variants detected by the MMCA assay.

	Reaction A		Reaction B		Reaction C		Reaction D
FAM	c.35delG		c.919‐2A>G		c.1707 + 5G>A		m.1555A>G
	5.19 ± 0.76		5.42 ± 1.34		6.1 ± 1.52		4.44 ± 1.09
HEX	c.167delT	c.176_191del16	c.2162C>T	c.2168A>G	c.1975G>C		m.1494C>T
	5.6 ± 1.15	9.22 ± 0.51	4.57 ± 1.65	−4.54 ± 1.48	−7.84 ± 0.58		8.1 ± 0.56
ROX	c.538C>T	c.547G>A	c.1174A>T		c.749T>C	c.754T>C	–
	9.02 ± 0.64	6.05 ± 0.5	6.05 ± 0.73		5.1 ± 0.97	−4.26 ± 1.33	–
ROX‐2	c.235delC		–		–		–
	4.82 ± 0.93		–		–		–
Cy5	c.299_300delAT		c.1226G>A	c.1229C>T	c.2027T>A		–
	−6.97 ± 0.87		−4.51 ± 1.97	7.0 ± 0.57	−6.5 ± 2.01		–

*Note*: Range (ΔTm ± 3SD, °C); ΔTm = Tm (wild‐type) − Tm (mutant); −, difference within ±1°C.

Abbreviations: *GJB2*, gap junction beta‐2; *GJB3*, gap junction beta‐3; *MT‐RNR1*, mitochondrial deoxyribonucleic acid; *SLC26A4*, solute carrier family 26, member 4.

### 2.5 Procedure of follow‐up

To ensure the efficient dissemination of genetic screening results, the following procedures were implemented: (1) Individuals who passed the genetic screening would receive a message notifying their guardians of the results; (2) Individuals who did not pass the genetic screening would be informed of their results and provided with suggestions either through a phone call or in person. The information and suggestions would primarily focus on explaining the genetic modes of hereditary HL, symptoms of specific types of HL, precautions, and prevention of risk factors (for carriers of *SLC26A4* and *MT‐RNR1* variants). The duration of the follow‐up would last for 1 year. For neonates diagnosed with HL, both the affected individuals and their parents would be advised to undergo genetic diagnosis to identify the genetic etiology, which would enable them to make informed medical decisions (Xie et al., 2021).

### 2.6 Statistical analysis

We performed the analysis with STATA 17.0. *χ* ^2^ tests and Fisher's precision tests were performed to assess the statistical significance of differences for categorical variables. All reported *p* values were 2‐tailed, and the significance threshold was defined as *p* < 0.05. Sequencing analysis was based on Vector NTI, and the reference sequence for comparison was cited from NCBI Reference Sequences (www.ncbi.nlm.nih.gov).

## 3 RESULTS

### 3.1 Variant frequency

**FIGURE 3:**
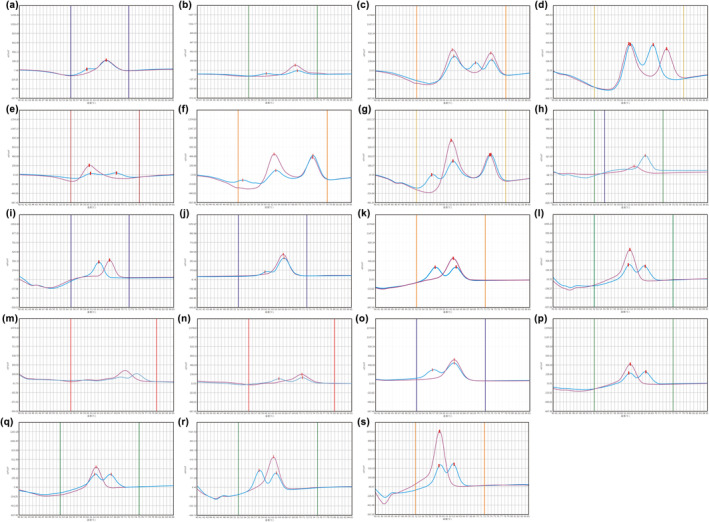
Melting curves of all HL‐associated variants detected in the study. Genotyping results of HL‐associated variants by the MMCA assay. In each pic, assorted colors of vertical lines on the background mean corresponding detection channels. Deep blue line means reaction A. Green line means reaction B. Yellow line means reaction C. Red line means reaction D. Purple line means positive control (wild type). Blue line means experimental group. (a) Heterozygous variant of c.35delG. (b) Heterozygous variant of c.176_191del16. (c) Heterozygous variant of c.235delC. (d) Homozygous variant of c.235delC. (e) Heterozygous variant of c.299_300delAT. (f) Heterozygous variant of c.538C>T. (g) Heterozygous variant of c.547G>A. (h) Homogeneous variant of m.1494C>T. (i) Homogeneous variant of m.1555A>G. (j) Heterozygous variant of c.919‐2A>G. (k) Heterozygous variant of c.1174A>T. (l) Heterozygous variant of c.1226G>A. (m) Homozygous variant of c.1226G>A. (n) Heterozygous variant of c.1229C>T. (o) Heterozygous variant of c.1707+5G>A. (p) Heterozygous variant of c.2168A>G. (q) Heterozygous variant of c.2027T>A. (r) Heterozygous variant of c.2162C>T. (s) Heterozygous variant of c.754T>C.

Among the participants, all variants were detected in the cohort except for three variants: *GJB2* c.167delT, *SLC26A4* c.1975G>C, and *SLC26A4* c.749T>C. Totally, the study found that 6.8% (170/2488) of all neonates carried at least one variant. Based on this, the allele frequency of HL‐associated variants in Jiangsu province was determined to be 3.7%. Figure 3 displays melting curves of all detected variants corresponding to the probes used for variants detection in the 4 different reaction types mentioned in Figure 2. The allele frequencies of the variants detected in the study are as follows: c.35delG (0.10%), c.176_191del16 (0.16%), c.235delC (1.15%), c.299_300delAT (0.28%), c.538C>T (0.18%), c.547G>A (0.08%), m.1494C>T (0.02%), m.1555A>G (0.18%), c.919‐2A>G (0.76%), c.1174A>T (0.04%), c.1226G>A (0.10%), c.1229C>T (0.10%), c.1707+5G>A (0.08%), c.2168A>G (0.16%), c.2027T>A (0.04%), c.2162C>T (0.04%), and c.754T>C (0.04%), and the results were presented in Table 2. Majority of the positive results were found in the *GJB2* gene, accounting for 50.0% (85 out of 170) of all carriers. Additionally, five neonates were found to carry double heterozygotes of *GJB2* or *SLC26A4*, including combinations of *GJB2* c.176_191del16 and *SLC26A4* c.919‐2A>G, *GJB2* c.235delC and *SLC26A4* c.919‐2A>G, *GJB2* c.176_191del16 and *GJB2* c.235delC, *GJB2* c.235delC and *GJB3* c.754T>C, *GJB2* c.299_300delAT and *SLC26A4* c.919‐2A>G. Three neonates were found carriers of homozygous variants, including one neonate with *GJB2* c.235delC, two neonates with *SLC26A4* c.1226G>A. All cases of *MT‐RNR1* variants discovered in the study were heterogeneous. Compared to the large‐scale study launched before, our study detected nine additional variants of *SLC26A4* (c.2162C>T, c.1174A>T, c.1226G>A, c.1707+5G>A, c.1975G>C, c.749T>C, c.754T>C, c.2027T>A, and c.1229C>T), which increased accuracy of *SLC26A4* inspection by 30.3% (20 out of 66) (Dai et al., 2019).

**TABLE 2:** Result of all variants in genetic screening.

Gene	Mutation	dbSNP	Classification of variants^a^	Number of carriers	Allele frequency (%)
*GJB2*	c.35delG	rs80338939	Pathogenic	5	0.10
	c.176_191del16	rs750188782	Pathogenic	8	0.16
	c.235delC	rs80338943	Pathogenic	58	1.17
	c.299_300delAT	rs111033204	Pathogenic	14	0.28
	c.167delT	rs80338942	Pathogenic	0	0.00
*GJB3*	c.538C>T	rs74315319	Uncertain significance	9	0.18
	c.547G>A	rs74315318	Conflicting interpretations of pathogenicity	4	0.08
*MT‐RNR1*	m.1494C>T	rs267606619	Drug response	1	0.02
	m.1555A>G	rs267606617	Drug response	9	0.18
*SLC26A4*	c.919‐2A>G	rs111033313	Pathogenic	39	0.78
	c.1174A>T	rs201562855	Pathogenic	2	0.04
	c.1226G>A	rs111033305	Pathogenic	3	0.10
	c.1229C>T	rs111033220	Pathogenic	5	0.10
	c.1707+5G>A	rs192366176	Pathogenic	4	0.08
	c.1975G>C	rs200455203	Pathogenic	0	0.00
	c.2168A>G	rs121908362	Pathogenic	8	0.16
	c.2027T>A	rs111033318	Pathogenic	2	0.04
	c.2162C>T	rs121908363	Pathogenic	2	0.04
	c.754T>C	NA	Pathogenic	2	0.04
	c.749T>C	rs1790985775	Uncertain significance	0	0.00

^a^ Classification was based on American College of Medical Genetics and Genomics (ACMG) guidelines for interpreting sequence variants (Oza et al., 2018).

**FIGURE 4:**
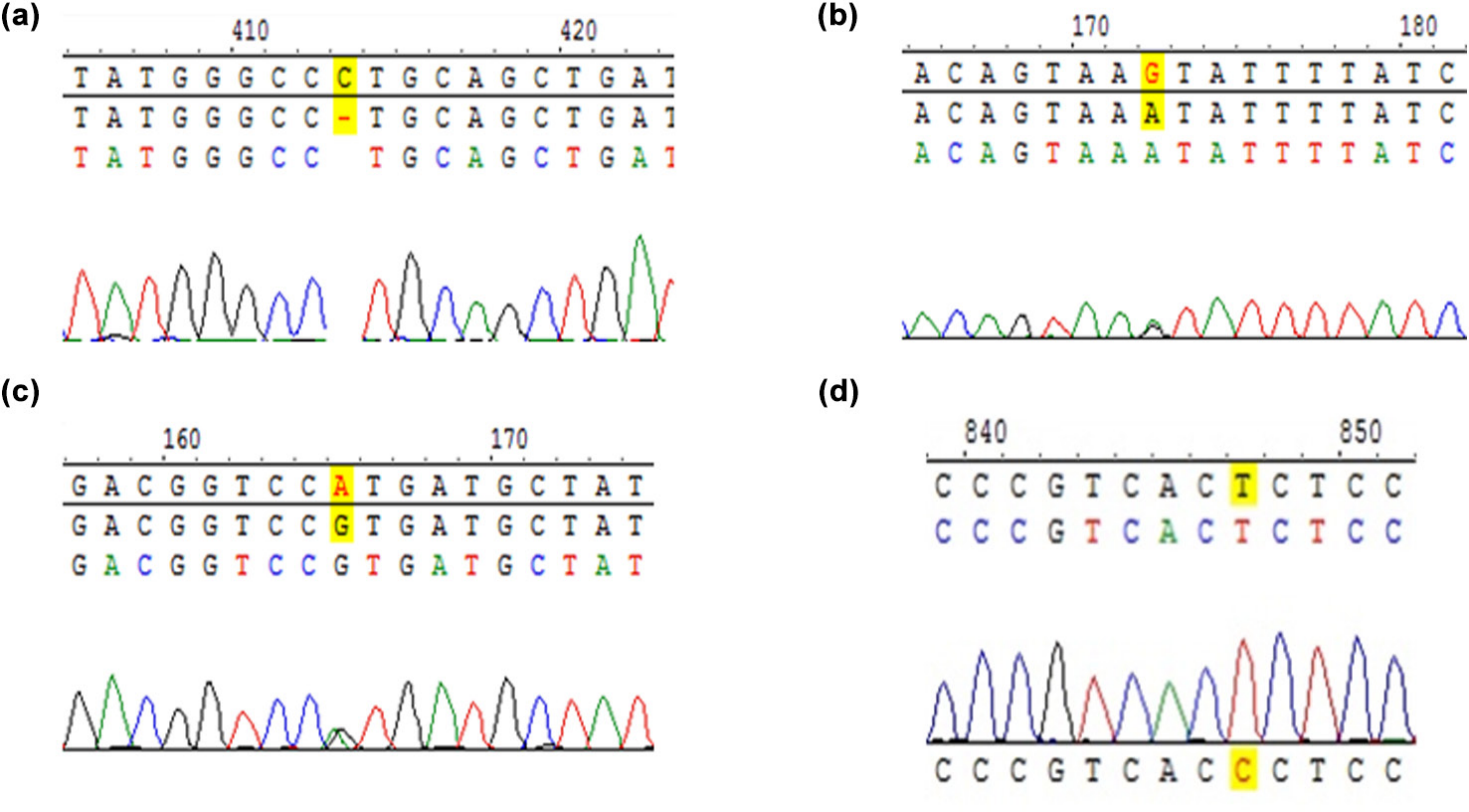
Sanger sequencing results of neonates referred in genetic screening. (a) Represents homozygous variant of *GJB2* c.235delC. (b) Represents homozygous variant of *SLC26A4* c.1226G>A. (c) Represents variant of m.1555A>G. (d) Represents variant of m.1494C>T.

All positive results accorded with Sanger sequencing results by Zeasan company. Part of sequencing results were shown in Figure 4, which were from referred neonates.

### 3.2 Epidemiology in Jiangsu province

**FIGURE 5:**
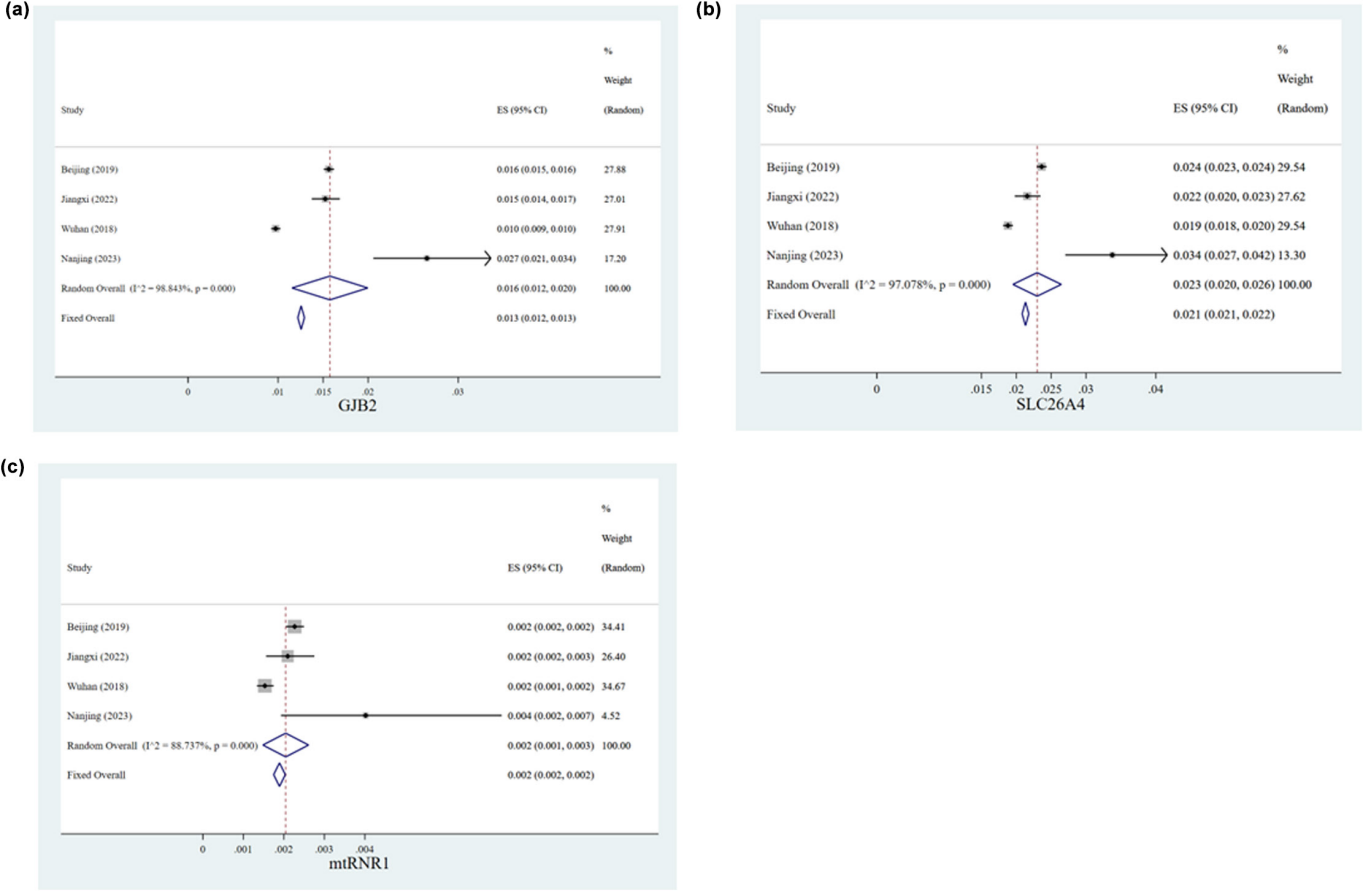
Tree diagram of meta‐analysis of studies about HL‐associated variants in different provinces in China. Discrepancy of three variants between regions was calculated under the framework of random effect model. (a) Distribution of *GJB2* variant; (b) distribution of *SLC26A4* variant; and (c) distribution of *MT‐RNR1* variant.

We made a meta‐analysis to expound the difference of genetic variant spectrum in different provinces in China. Three previous typical studies of common genetic screening of hearing loss among neonates in different areas of China were reviewed and three tree diagrams were shown in Figure 5 (Dai et al., 2019; Hao et al., 2018; Luo et al., 2022). Our findings revealed that Jiangsu province had a significantly higher carrier rate of *GJB2* variants, *SLC26A4* variants, and *MT‐RNR1* variants than that in other provinces of China (*p* < 0.05).

### 3.3 Correlation between hearing screening and genetic screening

Out of all the neonates, 144 were referred for further testing based on the OAE test, which accounted for 6.1% of the total. Thirty‐two neonates failed the second screening 6–12 weeks after birth, including seven neonates who passed the initial hearing screening but were unable to pass the second. It is worth mentioning that one of these neonates had a history of ototoxic drug use and was diagnosed with moderate HL, despite passing genetic screening with negative result. Among the neonates who failed the initial hearing screening, it was found that 18 of them carried HL‐related variants. The proportion of these variants was significantly higher compared to the group that had negative results in genetic screening (*p* = 0.014). Additionally, among the neonates who were positive in the second hearing screening, nine of them screened positive in genetic screening, showing a higher proportion of referral compared to the normal group (5.4% vs. 1.2%, *p* < 0.05). Our study identified 10 carriers of *MT‐RNR1* variants and 66 carriers of *SLC26A4* variants. Interestingly, all of them passed the hearing screening and had normal performance during follow‐up (Table 3).

**TABLE 3:** Genetic and hearing screening results of all neonates with positive genetic findings.

Gene	Variant	Zygosity	Hearing screening					Hearing diagnosis			
			Total	Initial		Second		Degree			Laterality
				Pass	Refer	Pass	Refer	M/M	S/P	Total	
*GJB2*	c.35delG	Het	5	4	1	5	0	NA	NA	0	
	c.176_191del16	Het	6	5	1	5	1	1	0	1	Unilateral
	c.235delC	Het	53	49	4	51	2	0	1	1	Bilateral
	c.235delC	Hom	1	0	1	0	1	0	1	1	Bilateral
	c.235delC/c.176_191del16	Het/het	1	1	0	0	1	0	1	1	Bilateral
	c.299_300delAT	Het	13	12	1	13	0	NA	NA	0	
*GJB3*	c.538C>T	Het	9	9	0	9	0	NA	NA	0	
	c.547G>A	Het	4	4	0	4	0	NA	NA	0	
*MT‐RNR1*	m.1494C>T	Het	1	1	0	1	0	NA	NA	0	
	m.1555A>G	Het	9	9	0	9	0	NA	NA	0	
*SLC26A4*	c.919‐2A>G	Het	35	33	2	33	2	0	1	1	Unilateral
	c.1174A>T	Het	2	2	0	2	0	NA	NA	0	
	c.1226G>A	Het	1	1	0	1	0	NA	NA	0	
	c.1226G>A	Hom	2	1	1	0	2	1	1	2	Bilateral/bilateral
	c.1229C>T	Het	5	4	1	5	0	NA	NA	0	
	c.1707+5G>A	Het	4	4	0	4	0	NA	NA	0	
	c.2168A>G	Het	8	7	1	8	0	NA	NA	0	
	c.2027T>A	Het	2	2	0	2	0	NA	NA	0	
	c.2162C>T	Het	2	2	0	2	0	NA	NA	0	
	c.754T>C	Het	1	1	0	1	0	NA	NA	0	
Other	c.176_191del16/c.919‐2A>G	Het/het	1	1	0	1	0	NA	NA	0	
	c.235delC/c.919‐2A>G	Het/het	1	0	1	1	0	NA	NA	0	
	c.235delC/c.754T>C	Het/het	1	1	0	1	0	NA	NA	0	
	c.299_300delAT/c.919‐2A>G	Het/het	1	1	0	1	0	NA	NA	0	

Abbreviations: *GJB2*, gap junction beta‐2; *GJB3*, gap junction beta‐3; Het, heterozygosity; Hom, homozygosity; M/M, mild or moderate degree of hearing loss; *MT‐RNR1*, mitochondrial deoxyribonucleic acid; NA, not applicable; NHS, newborn hearing screening; S/P, severe or profound degree of hearing loss; *SLC26A4*, solute carrier family 26, member 4.

### 3.4 Outcomes of neonates

Ultimately, 11 neonates were diagnosed with HL, resulting in a prevalence of 4.4 per 1000 infants of HL in Jiangsu. Among neonates diagnosed with HL, seven of them carried at least one variant in Table 4. We detected *SLC26A4* c.1226G>A in three carriers, with two carriers having a homozygous variant and both were diagnosed as HL. It is important to note that all participants who were referred for genetic screening were found to have bilateral HL.

**TABLE 4:** Overview of hearing loss cases identified in the study.

Subject	Sex	Age of diagnosis (month)	NHS	Seriousness	Laterality	Gene	Mutations	Treatment
1	Male	11	Pass	S/P	Bilateral	*GJB2*	c.235delC/c.176_191del16	2
2	Female	9	Fail	S/P	Bilateral	*GJB2*	c.235delC het	2
3	Female	9	Fail	M/M	Unilateral	NA	NA	3
4	Male	7	Fail	S/P	Unilateral	*SLC26A4*	c.919‐2A>G het	3
5	Female	12	Pass	M/M	Bilateral	NA	NA	2
6	Female	7	Fail	S/P	Unilateral	NA	NA	3
7	Male	8	Fail	M/M	Unilateral	NA	NA	3
8	Male	10	Pass	S/P	Bilateral	*SLC26A4*	c.1226G>A hom	1
9	Male	6	Fail	S/P	Bilateral	*GJB2*	c.235delC hom	1
10	Female	7	Fail	M/M	Bilateral	*SLC26A4*	c.1226G>A hom	3
11	Male	6	Fail	M/M	Unilateral	*GJB2*	c.176_191del16 het	3

*Note*: Treatment: 1, cochlear implants; 2, hearing aid; 3, rehabilitation therapy and speech therapy.

Abbreviations: Het, heterozygosity; Hom, homozygosity; M/M, mild or moderate degree of hearing loss; NA, not applicable; S/P, severe or profound degree of hearing loss.

For carriers who passed hearing screening with heterozygous variants of *GJB2* or *SLC26A4*, it was still recommended to proceed with the follow‐up of hearing to take precautions against delayed‐onset HL. The results of genetic screening also had significance in tracing the reasons of genetic deafness and prospective counseling in premarital examination. As was reported before, there was no statistical difference between GJB3 c.538C>T variant carrier and normal, so carriers with GJB3 variants received follow‐up without additional intervention after passing hearing screening in our study (Huang et al., 2017). Carriers with variants of *MT‐RNR1* were suggested to avoid ototoxic drugs or use the drugs prescribed by qualified medical staff. Additionally, carriers' variants of *MT‐RNR1* usually inherited from maternal mitochondrial genes, which also indicated that their matrilineal family members were also in considerable risk of HL.

## 4 DISCUSSION

Nowadays, UNHS is implemented worldwide and has achieved significant success in preventing speech and language developmental delays in neonates with HL (Tobe et al., 2013). Considering that not all phenotypes of genes related to HL manifest at birth, UNHS has limitations. The majority of cases of genetic HL are due to autosomal recessive inheritance, making them difficult to predict as in 75%–80% of cases, in which both parents of newborns with HL have normal hearing and may neglect the possibility of genetics being involved (Kochhar et al., 2007; Smith et al., 2005). Combining hearing screening with genetic screening can help identifying neonates with underlying HL, not only because their symptoms may appear late and even more severe if misdiagnosed in hearing screening (Zhu et al., 2022), but also because the results might well make sense in the tertiary prevention of HL. By clinical follow‐up, two neonates with variants of GJB2 and SLC26A4 were diagnosed as HL after passing the initial hearing screening, which was consistent with the conclusion that genetic screening increased the accuracy of hearing screening alone (Q. Wang, Xiang, et al., 2019). Cochlear implantation for HL patients with *GJB2* or *SLC26A4* variants before 3.5 years will lead to favorable prognosis, early detection of variants in *MT‐RNR1* makes drug‐induced HL preventable (Kochhar et al., 2007; Wang, Ding, et al., 2019; Wu et al., 2015). Our study showed that the genetic and hearing screening program allowed for the identification of a targeted population (2.9%; 72 out of 2488) that required referral for specialized care and early intervention.

Currently, there have been 124 genes identified as being associated with non‐syndromic HL (http://hereditaryhearingloss.org). Furthermore, the complexity of the variant spectrum is compounded by variations in ethnic backgrounds, making statistical analysis challenging. As was reported in a meta‐analysis, the *GJB2* c.235delC variant did not significantly correlate with non‐syndromic HL among Oceania and European populations but played an important role in cases of HL among populations of East Asian and Southeast Asian descent (Yao et al., 2012). Additionally, *GJB2* c.167delT was identified as a hotspot variant among the Ashkenazi Jewish population, with a high carrier frequency of 4% (Putcha et al., 2007). But contrary to that in our study, no neonates were discovered the carrier of this variant (0%). *GJB3* c.538C>T and *GJB3* c.547G>A were originally found to eventuate bilateral high‐frequency hearing impairment. However, recent studies found that *GJB3* c.538C>T had a low prevalence in China and compared HL cases with normal hearing cases, carrier frequency of *GJB3* c.538C>T differed not significantly (0.38%:0.24%, *p* > 0.05) (Chen et al., 2014; Huang et al., 2017). *SLC26A4* gene encodes the anion exchanger pendrin, of which the variants will lead to non‐syndromic recessive HL and Pendred syndrome (Honda & Griffith, 2022; Kim et al., 2019). Variants of *SLC26A4* are the second most frequent causes of hereditary HL in China and prevalence of two common variants (c.919‐2A>G,c.2168 A>G) are 1.02% and 0.14% (Fu et al., 2019; Pan et al., 2022; Zhang et al., 2023). Hereditary HL attributed to *SLC26A4* gene is manifested as unique fluctuations and sudden drops of hearing induced by head impact. Different from other three genes screened in genetic HL, *SLC26A4* gene has a bigger size and 99% variants have been found in Chinese population (Danilchenko et al., 2022). It was illustrated in our study that except two common variants of *SLC26A4* (c.919‐2A>G,c.2168 A>G) which have been approved for genetic screening in China, c.1226G>A, c.1707+5G>A, and c.1229C>T should also be screened for higher accuracy.

In the study, we evaluated the analytical and clinical performances of MMCA when running the genetic screening. The MMCA assay can be performed using only a real‐time PCR thermocycler, which is a cost‐effective instrument commonly found in standard molecular biology laboratories (Wang et al., 2017). This eliminates the need for expensive equipment such as a sequencer or MS spectrometer to initiate the HL screening program, thereby reducing the expense and time cost of MMCA (approximately $5 per sample and 3–4 h per batch). Additionally, MMCA can expand its scope by a mode of detection of one channel and multiple genotypes realized by the combined use of multicolor real‐time PCR and melting curve analysis and might allow the use of a variety of sources of clinical samples for screening its wide range of template concentration.

## 5 CONCLUSIONS

To sum up, by analyzing the distribution of common HL‐associated variants among neonates, we identified a high carrier frequency of these variants in the population of Jiangsu province. Our study indicated that limited genetic screening could compensate the weakness of UNHS in identifying for potential HL and decrease medical cost concurrently. MMCA made large‐scale genetic screening attainable, in which we can evaluate the cost‐effectiveness and potential benefits by establishing deafness prevention strategies in the future.

## AUTHOR CONTRIBUTIONS

Lizhou Sun, Yi Liu and Jue Wang designed this study. Yuan Zhan, Huiyan Wang, and Qian Meng made contributions for collection of samples. Yi Liu and Shengnan Song performed the experiments. Yi Liu drafted the article. Yi Liu, Jue Wang collected the clinical data and follow‐up information. Yuanyuan Zhang and Yetao Xu revised the manuscript.

## CONFLICT OF INTEREST STATEMENT

All authors declare no conflict of interest.

## ETHICS STATEMENT

This study was approved by the ethics committees of Ethics Committee of Jiangsu province Hospital affiliated with Nanjing Medical University and all hospitals involved (2022‐SR‐049). Confidentiality of privacy would be ensured after written consents obtained from the parents of all neonates who were incorporated into the study cohort and collected information of hearing and habilitation.

## Supporting information


Table S1.

## Data Availability

The data that support the findings of this study are available from the corresponding author, [author initials], upon reasonable request.
