# Supplementary material for: Multicolor melting curve analysis discloses high carrier frequency of hearing loss‐associated variants among neonates in Jiangsu province
Source: Mol Genet Genomic Med. 2024 Feb 6;12(2):e2384. doi: 10.1002/mgg3.2384 (PMC10847707; doi:10.1002/mgg3.2384)
Supplement: Supplementary file 1 — Table S1. [file MGG3-12-e2384-s001.docx]

Table S1 20 hearing-loss associated variants detected by multicolor melting curve analysis

| Detection of Hearing-loss associated Genes | Variants of Corresponding Genes |
| --- | --- |
| GJB2 | c.35delG |
|  | c.176_191del16 |
|  | c.235delC |
|  | c.299_300delAT |
|  | c.167delT |
| GJB3 | c.538C>T |
|  | c.547G>A |
| mtRNR-1 | m.1494C>T |
|  | m.1555A>G |
| SLC26A4 | c.919-2A>G |
|  | c.1174A>T |
|  | c.1226G>A |
|  | c.1229C>T |
|  | c.1707+5G>A |
|  | c.1975G>C |
|  | c.2168A>G |
|  | c.2027T>A |
|  | c.2162C>T |
|  | c.754T>C |
|  | c.749T>C |
